# Tracheal Diverticulum in a Young Male Patient With Chronic Cough: A Case Report

**DOI:** 10.7759/cureus.36006

**Published:** 2023-03-10

**Authors:** Pugazhendi Inban, Sai Harini Chandrasekaran

**Affiliations:** 1 Department of General Medicine, Government Medical College Omandurar, Chennai, IND

**Keywords:** cystic lesion, asymptomatic, ct scan, air cyst, cough, tracheal diverticulum

## Abstract

A tracheal diverticulum is a rare clinical entity that is mostly diagnosed incidentally in radiographic pictures. It is infrequently mentioned in literature because of its relatively asymptomatic presentation. Its etiology is mostly attributed to tracheal wall weakness, which could either be congenital or acquired. Here we present a case of a 26-year-old man who presented with chronic intermittent cough and was incidentally diagnosed to have a tracheal diverticulum on the radiographic pictures of a high-resolution computed tomography scan of the chest (HRCT chest).

## Introduction

Tracheoceles, bronchogenic cysts, lymphoepithelial cysts, and tracheal diverticula are all conditions collectively referred to as paratracheal air cysts. They can appear in a variety of ways, ranging from being totally asymptomatic to some terrifying scenarios like pneumothorax and pneumoperitoneum. This can create a diagnostic conundrum.

The tracheal diverticulum can simply be described as a small air-filled invagination that can be observed in the paratracheal region of the neck. They can be single or numerous, and histologically these air-filled invaginations are found to be lined by ciliated columnar epithelium. Seldom seen in clinical settings, tracheal diverticula are typically found by accident during radiological imaging. Although it is a benign pathological condition, it has the ability to produce long-lasting symptoms such as chronic cough and can sporadically result in airway obstruction [1].

In this case report, we present a case of a 26-year-old man who was identified to have a tracheal diverticulum during the patient's evaluation for a chronic cough.

## Case presentation

A 26-year-old man working as a software engineer presented with a complaint of cough for 12 weeks. The cough was dry and intermittent in nature. The cough was not associated with blood streaks and there was no history of sputum production associated with the cough. There were no specific aggravating or relieving factors associated with the cough. The patient tried some home remedies such as drinking hot water and taking honey during episodes without any symptomatic improvement. The patient did not have any other systemic symptoms such as fever, chest pain, or breathing difficulty. There were no histories suggestive of gastroesophageal reflux disease (GERD) and postnasal drip. The patient did not have any contact history or symptoms suggestive of tuberculosis (TB). The patient is up to date on vaccinations and does not have any significant past medical history including allergic history. He has smoked since the age of 18 but was otherwise healthy without any significant personal or treatment history.

Physical examination and a detailed respiratory system examination including lung auscultation did not reveal any significant abnormality. Blood investigations including hemogram, erythrocyte sedimentation rate (ESR), and C-reactive protein (CRP) demonstrated normal values. Interferon-gamma release assay (IGRA) for TB (QuantiFERON-TB Gold Test) gave negative results. Spirometry was done for the patient and the parameters were normal, suggesting the absence of any obstructive or restrictive lung disorder.

**Figure 1:**
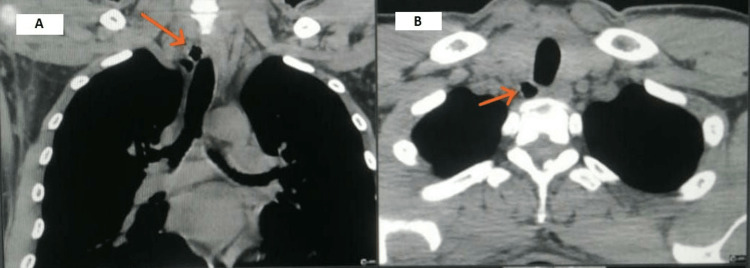
A - CT chest (mediastinal view) showing a cystic pouch connected to the tracheal lumen with a thin stalk. B - CT chest (axial view) showing a tracheal diverticulum in the paratracheal region. CT: computed tomography

A high-resolution computed tomography (HRCT) scan of the chest demonstrated a small cystic lesion near the trachea on its postero-lateral aspect (Figures 1A-1B). The CT scan also revealed that the cystic lesion was connected to the tracheal lumen with a very thin stalk (Figure 1A). This cystic lesion was concluded to be a tracheal diverticulum based on the typical location of the lesion and its connection to the tracheal lumen with a slender stalk. The CT scan of the chest was otherwise normal without any parenchymal pathology.

Bronchoscopy was done to study any pathology related to the tracheal surface and it was normal. Bronchoalveolar lavage (BAL) was also carried out along with bronchoscopy and the results were inconclusive of any infective or malignant pathology.

Based on the normal white blood cell counts, normal ESR, normal CRP, absence of significant systemic symptoms, negative IGRA for TB, and a normal BAL analysis - infection was ruled out as a possible cause for the symptom. Other potential causes of cough such as GERD, postnasal drip, and allergy were ruled out from the patient's history. Spirometry was normal and the CT scan of the chest did not reveal any other pathology other than the tracheal diverticulum ruling out other lung parenchymal disorders as a cause for the cough. Based on these findings, it was collectively concluded that the patient's cough could be due to the tracheal diverticulum.

The patient was managed conservatively. He was counseled about smoking cessation and was treated with cough suppressant lozenges and syrup. He was asked to follow up after a period of two months or earlier if the symptoms persist or worsen.

During the follow-up after two months, the patient remained healthy and presented with a resolution of cough.

## Discussion

Tracheal diverticula have a reported incidence of 2.0% and a prevalence that ranges from 0.75% to 8.1%. The most common location of these diverticula is found to be the right posterolateral (98.5%) and less frequently on the left side (1.5%) at the level of T1-T3 vertebrae [2]. Even though most of the cases are asymptomatic, if the diverticulum is large, it can produce symptoms like dry cough, pain, shortness of breath, and dysphonia. If left untreated, large tracheal diverticula can lead to repeated infections due to secretions and can also complicate intubation and assisted ventilation [3].

Radiologists have recently observed that the number of tracheal diverticulum cases is increasing trend. A tracheal diverticulum can be categorized into two types: congenital diverticulum and acquired diverticulum based on the etiology and pathological process [4]. Congenital diverticulum results from either a deficiency in tracheal cartilage development during the sixth week of fetal life or a defect in endodermal differentiation during the development of the membranous posterior tracheal wall [5]. The congenital diverticulum has a full tracheal anatomy, including smooth muscle, cartilage, and respiratory epithelium, and is typically mucus-filled. The trachea-esophageal fistula and other congenital abnormalities might occasionally coexist with it [6].

Although acquired tracheal diverticula can develop at any level, the poster lateral area is where they most frequently occur. Congenital tracheal diverticula are smaller and have a narrower orifice than acquired tracheal diverticula. One or several acquired tracheal diverticula are possible. They develop as a result of increasing intraluminal pressure, which causes the mucous membrane to herniate through a weak spot in the tracheal wall and is accompanied by a persistent cough, such as in chronic obstructive pulmonary disease. Contrary to congenital diverticula, acquired tracheal diverticula have a wall made entirely of respiratory epithelia and no cartilage or smooth muscle.

The diagnosis of this uncommon condition can be established using a CT scan of the trachea (ideally a spiral or helical CT) and re-construction from various angles in the coronal plane to see communication with the tracheal wall. Based on the presence or absence of cartilage and the size of the diverticulum's neck, CT can help distinguish between congenital and acquired lesions by providing information on the location, origin, and size of the lesion [7]. Further progress in the morphological diagnosis of this entity may benefit from the creation of novel methods like three-dimensional reconstruction. There are reports that support the discovery of a tracheal diverticulum during surgery or an autopsy. Diverticula with a small opening or only a fibrous link to the trachea can be missed by bronchoscopy even though it can confirm the diagnosis [8,9].

Treatment options include endoscopic cauterization with laser or electrocoagulation, surgical excision, which can be carried out by a lateral cervical route without the necessity for a thoracotomy, and conservative care (antibiotics, mucolytics, and physical therapy). However, the treatment options mentioned are considered only when the diverticulum is identified as the cause of symptoms in patients. Excision surgery has reportedly shown excellent outcomes. The choice of treatment relies on the patient's symptoms, age, and physical condition, with older patients, often benefiting more from conservative management and younger people generally benefiting more from surgery [10,11].

## Conclusions

In conclusion, the tracheal diverticulum is an uncommon clinical condition diagnosed incidentally in radiographic images. Usually, these tracheal diverticula are not associated with significant symptoms. However, if the diverticulum is large, there is always a possibility that it could lead to clinical complications requiring intervention. A fast and reliable diagnosis of the tracheal diverticulum may be made with the aid of diagnostic tools like HRCT and 3D reconstruction technologies. Treatment options for symptomatic patients depend on patient factors and co-morbidities. Symptomatic patients may be managed with conservative measures. Surgical options could be considered only when conservative measures fail as there is always an increased risk of neurovascular complications occurring with these surgical options.
